# Implementing alternatives to coercion as a key component of improving mental health care: the WPA contribution

**DOI:** 10.1192/j.eurpsy.2023.111

**Published:** 2023-07-19

**Authors:** S. Galderisi

**Affiliations:** University of Campania “Luigi Vanvitelli”, Naples, Italy

## Abstract

**Abstract:**

To favor the implementation of alternatives to coercive practices, a WPA Taskforce and reference group on Minimizing Coercion in Mental Health Care was created within the WPA 2017-2020 Action Plan. It included several distinguished colleagues from different countries and cultural as well as experiential background, and representatives from patients and carers organizations. Task force members soon realized the presence of a significant diversity of views and experiences among mental health professionals, people with lived experience and their carers. All members agreed that the debate on minimizing versus eliminating coercion could be endless and unfruitful, while the opportunity to concentrate on improving the quality of mental health care in low-, middle- and high-income countries, and implementing alternatives to coercion as a key component of improving mental health care, was instead a shared goal that could make the task of the group feasible and productive. The General Assembly of the WPA in October 2020 approved a Position statement drafted by the Task Force aimed to set a direction and practical starting point for action. This presentation will illustrate contribution provided by the WPA Working Group for Implementing Alternatives to Coercion in Mental Health Care within the current WPA Action Plan (2020-2023).

**Disclosure of Interest:**

None Declared

